# Electrocorticographic representations of segmental features in continuous speech

**DOI:** 10.3389/fnhum.2015.00097

**Published:** 2015-02-24

**Authors:** Fabien Lotte, Jonathan S. Brumberg, Peter Brunner, Aysegul Gunduz, Anthony L. Ritaccio, Cuntai Guan, Gerwin Schalk

**Affiliations:** ^1^Inria Bordeaux Sud-Ouest/LaBRITalence, France; ^2^Department of Speech-Language-Hearing, University of KansasLawrence, KS, USA; ^3^National Center for Adaptive Neurotechnologies, Wadsworth Center, New York State Department of HealthAlbany, NY, USA; ^4^Department of Neurology, Albany Medical CollegeAlbany, NY, USA; ^5^J. Crayton Pruitt Family Department of Biomedical Engineering, University of FloridaGainesville, FL, USA; ^6^A^*^STAR Agency for Science, Technology and Research, Institute for Infocomm Research, SingaporeSingapore

**Keywords:** electrocorticography (ECoG), speech processing, place of articulation, manner of articulation, voicing

## Abstract

Acoustic speech output results from coordinated articulation of dozens of muscles, bones and cartilages of the vocal mechanism. While we commonly take the fluency and speed of our speech productions for granted, the neural mechanisms facilitating the requisite muscular control are not completely understood. Previous neuroimaging and electrophysiology studies of speech sensorimotor control has typically concentrated on speech sounds (i.e., phonemes, syllables and words) in isolation; sentence-length investigations have largely been used to inform coincident linguistic processing. In this study, we examined the neural representations of segmental features (place and manner of articulation, and voicing status) in the context of fluent, continuous speech production. We used recordings from the cortical surface [electrocorticography (ECoG)] to simultaneously evaluate the spatial topography and temporal dynamics of the neural correlates of speech articulation that may mediate the generation of hypothesized gestural or articulatory scores. We found that the representation of place of articulation involved broad networks of brain regions during all phases of speech production: preparation, execution and monitoring. In contrast, manner of articulation and voicing status were dominated by auditory cortical responses after speech had been initiated. These results provide a new insight into the articulatory and auditory processes underlying speech production in terms of their motor requirements and acoustic correlates.

## 1. Introduction

Speech and language are realized as acoustic outputs of an aeromechanical system that is coordinated by a vast brain and muscular network. The interaction between neural structures, facial and vocal tract musculature, and respiration provides humans with a dynamic speech production system capable of forming simple sounds (e.g., mono-syllabic words) and complex sounds (e.g., fluent conversation). These sounds are often represented by phonemes and syllables, which are fundamental linguistic bases for constructing both simple and complex speech production (e.g., the “b” in “bad” is an example of a phoneme while the “ba” is an example of a consonant-vowel (CV) syllable), which in turn correspond to stereotyped vocal-tract movements resulting in acoustic speech output. Examples of such vocal-motor articulations range from the compression of the lungs for producing the air pressure needed for vocalization, to movements of laryngeal muscles during phonation, to configurations of the upper vocal tract for final shaping of speech output. These muscular actions are the behavioral consequences of the speech neuromotor system, which is in turn driven by phonological constructs and lexical relationships (Indefrey and Levelt, 2004).

This type of communication relies on neural processes that construct messages and sensorimotor commands to convey and receive communicative information. These processes have previously been characterized in a theoretical neurolinguistic model, the Levelt-Roelofs-Meyer (LRM) model (Levelt et al., 1999). Using this model as a framework, it is possible to investigate the behavioral, neurological, linguistic and motor processes involved in vocal communication. The model consists of the following processing components: conceptual preparation, lexical selection, morpho-phonological code retrieval, phonological encoding, phonetic encoding and articulation (Levelt et al., 1999; Indefrey and Levelt, 2004). The first four processing levels in the LRM framework all mediate perceptual processes underlying speech and language recognition in preparation for upcoming vocal productions (e.g., reading, picture naming). These levels of processing have been well investigated and were summarized in a meta-analysis of neuroimaging, electrophysiology and neuro-stimulation studies of speech and language (Indefrey and Levelt, 2004), and more recently by Riès et al. (2013). The final two stages, phonetic encoding and articulation (of articulatory scores), describe the motor aspects of vocal communication and are the focus of the present study. According to the LRM framework, the phonetic encoding stage translates a phonological word (from the previous phonological encoding stage) into an articulatory score, which can be processed and transmitted to the articulatory musculature for speech motor output.

The precise nature by which the brain realizes these phonetic encoding and articulation functions are still unknown. One possible explanation for this lack of understanding stems from the difficulty in measuring the neurological processes involved in the planning and production of speech. Indefrey and Levelt estimate a total speech-language processing time of approximately only 600 ms (not including articulation) from beginning to end, with individual durations of approximately 100–200 ms for each processing component in their model (Indefrey and Levelt, 2004). Functional magnetic resonance imaging (fMRI), which is the primary neuroimaging technique used in speech neuroscience, cannot resolve brain activity at that temporal resolution. In contrast, electroencephalography (EEG) and magnetoencephalography (MEG) can readily detect neurological signals at these temporal scales, but cannot precisely ascribe their source to a particular location. In addition, EEG, MEG, and fMRI are all susceptible to electrical and/or movement artifacts created by speech articulation, and thus are typically used to investigate neurological activity prior to articulation or speech perception.

Electrical signals recorded directly from the cortical surface [electrocorticography (ECoG)] have recently begun to attract increasing attention for basic and translational neuroscience research, because they allow for examination of the precise spatio-temporal evolution of neurological processes associated with complex behaviors, including speech output. Specifically, ECoG has been used to investigate neurological activity during a number of tasks including linguistic processing (Towle et al., 2008; Edwards et al., 2010), speech perception and feedback processing (Crone et al., 2001; Chang et al., 2010; Pei et al., 2011a,b; Pasley et al., 2012), as well as articulation of phonemes, syllables, and words (Blakely et al., 2008; Kellis et al., 2010; Leuthardt et al., 2012; Bouchard et al., 2013; Riès et al., 2013; Mugler et al., 2014). In the present study, we apply machine learning techniques to evaluate the neurological activity during speech production based on segmental features (i.e., phonology, and articulatory-acoustic descriptors) and the resulting ECoG signals. By analyzing these features, rather than phonemes, syllables, or words, we are able to identify a low-dimensional and invariant basis by which to interpret neural activity related to overt speech production that can be upscaled to more complex vocalizations.

A recent ECoG study (Bouchard et al., 2013) employed such an articulation-based approach in which subjects were required to produce isolated CV syllables. The authors observed both a topographic and temporal organization of ECoG signals over the speech-motor cortex related to speech articulation. Specifically, their results showed that the production of isolated syllables resulted in differential neurological activity clustered by articulatory feature (e.g., lip and tongue movements). These findings greatly contributed to our understanding of the motor cortical representations of isolated syllable production; however, in typical speech, syllable production is rarely performed in isolation. Here, we generalize and improve upon these results by investigating articulation as it occurs during continuous, fluent speech. One major difference between isolated production of speech sounds and continuous speech is the presence and degree of coarticulation, or the influence of past and future speech requirements over current productions (Hardcastle and Hewlett, 1999). The two varieties of coarticulation include: (1) carry-over, in which upcoming speech productions are based on the vocal tract configurations of past utterances; and (2) anticipatory, in which the production of current speech sounds is altered based on expected requirements of future sounds. The extent to which segmental and phonological boundaries influence the degree of coarticulation (Recasens, 1999) is currently subject of debate (e.g., whether a boundary facilitates or inhibits coarticulation). In our study, we assume coarticulation is occurring as participants produce speech, and our results are based solely on the amount of speech information present in the ECoG signal.

In our experiments, we asked subjects to perform an out-loud speech production task. We recorded the subjects' acoustic output with a microphone and ECoG from widespread perisylvian areas that included locations with known involvement in the planning, execution and perception of speech. For each subject, we then converted the subject's acoustic output into speech feature categories at the phonetic level (given in **Table 2**) and applied machine learning techniques to identify differential brain activity resulting from the production of specific speech features. The features used in our work were: place of articulation, manner of articulation, voicing status and phonological category of consonant or vowel. These techniques allowed us to investigate the topographical as well as temporal distributions of brain activity that differentiates each type of speech feature amongst other features, which may temporally overlap in continuous speech. The analysis techniques used in our study can also be used to predict the occurrence of a speech feature from the ECoG signals. Therefore, our study provides important insights into the coordination of individual articulatory neuromotor processes as they are sequenced together for production of fluent speech output, and should provide an important basis for future development of a brain-to-text brain-computer interface (BCI).

The results of our analyses revealed a broad network involving fronto-motor and temporal cortices that were active during the preparation, execution and feedback monitoring of place of articulation. In contrast, ECoG responses labeled by manner of articulation involved a widespread auditory cortical network that was active near the start of speech onset and persisting throughout the feedback monitoring process. Analysis of voicing status largely mirrored the manner of articulation results suggesting that the production of different manners of articulation and voicing involve large auditory cortical networks for processing for proper speech motor control, while place of articulation more equally weights processing at all three stages of production. Interestingly, our analysis of both the manner and voicing conditions included a focal motor response that likely reflects specific differences in the motor control of voicing (e.g., voiced vs. voiceless production). We elaborate on these results and their interpretation in the sections that follow.

## 2. Materials and methods

### 2.1. Human subjects and data collection

The seven subjects who participated in this study were patients with intractable epilepsy at Albany Medical Center. Subjects underwent temporary placement of subdural electrode arrays to localize seizure foci prior to surgical resection of epileptic tissue. All gave informed consent to participate in the study, which was approved by the Institutional Review Boards of the hospital, had performance IQs of at least 85, and were mentally, visually and physically capable of performing the task. Table 1 summarizes the subjects' clinical profiles.

**Table 1:** **Clinical profiles of participants**.

**Subject**	**Age**	**Sex**	**Handedness**	**Performance IQ**	**Verbal IQ**	**Seizure focus**	**# Electrodes**	**# Words**
A	29	F	R	136	118	Left temporal	96	278
B	30	M	R	90	64	Left temporal	83	109
C	29	F	R	90	91	Left temporal	101	283
D	19	M	R	85	87	Left frontal	84	411
E	26	F	R	117	106	Left temporal	109	411
F	56	M	R	87	82	Left temporal	97	411
G	29	F	R	95	111	Left temporal	112	411

The implanted electrode grids (Ad-Tech Medical Corp., Racine, WI) consisted of platinum-iridium electrodes (4 mm in diameter, 2.3 mm exposed) that were embedded in silicon and spaced at an inter-electrode distance of 1 cm. Subject G had implanted electrodes with 6 mm grid spacing (PMT Corp, Chanhassen, MN). All subjects received electrode grid implantations over the left hemisphere, though the total number of electrodes implanted was different for each subject. Grid placement and duration of ECoG monitoring were based solely on the requirements of the clinical evaluation without any consideration of this study.

**Figure 1:**
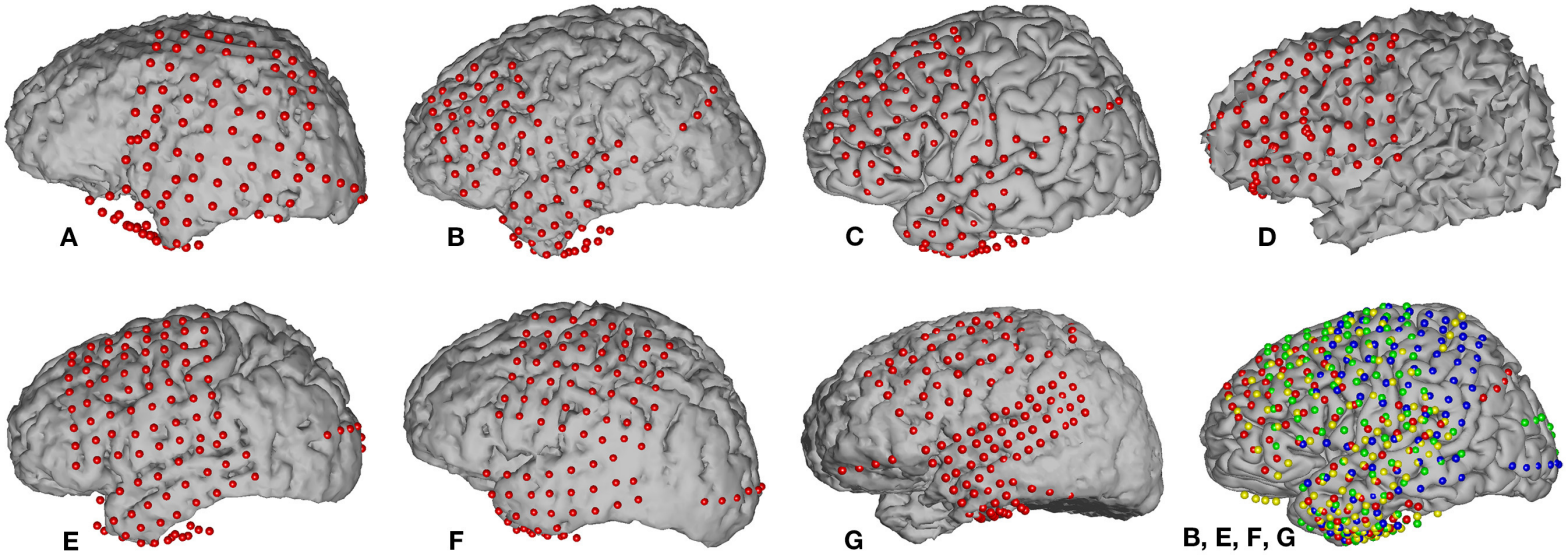
**Locations of implanted grids on individual subject cortical models based on co-registered pre-op MR and post-op CT data**. The bottom right figure shows the electrode locations projected on an average brain for those four subjects (**B, E, F**, and **G**) that passed initial screening (subjects **A**, **C**, and **D** were not included, see Section 2.3.5). Each subject's electrodes are represented with a different color.

Grid locations were verified in each subject using a co-registration method that included pre-operative structural magnetic resonance (MR) imaging and post-operative computed tomography (CT) imaging (Kubanek and Schalk, 2014). We then used Curry software (Compumedics, Charlotte, NC) to extract three-dimensional cortical models of individual subjects, to co-register the MR and CT images, and to extract electrode locations. Electrode locations are shown for each subject in Figure 1. Electrode locations were further assigned to cortical lobe using the Talairach Daemon (http://www.talairach.org, Lancaster et al., 2000).

ECoG signals were recorded at the bedside using eight 16-channel g.USBamp biosignal acquisition devices (g.tec, Graz, Austria) at a sampling rate of 1200 Hz, and stored for further analyses. Electrode contacts distant from epileptic foci and areas of interest were used for reference and ground and any channels with obvious electrical or mechanical artifacts removed. The total number of electrodes used per subject is listed in Table 1.

### 2.2. Experimental paradigm

**Figure 2:**
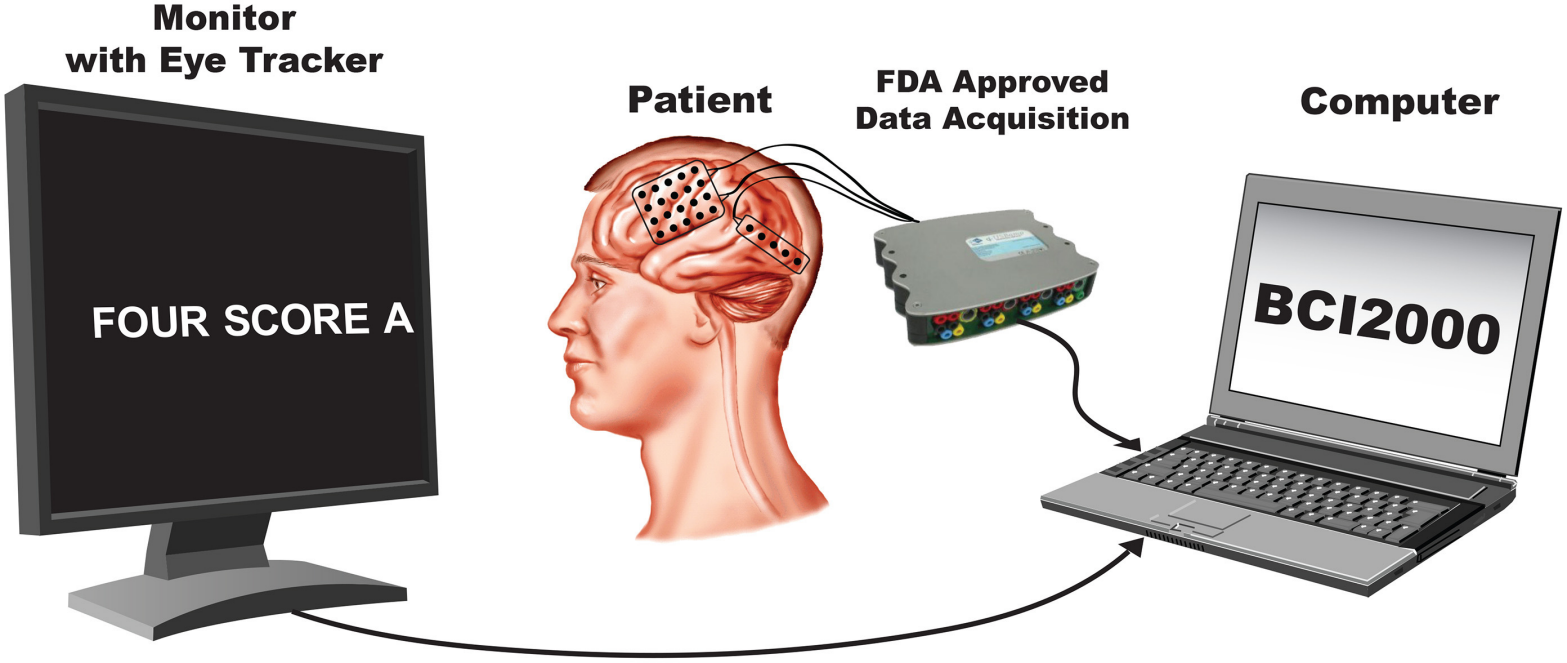
**Experimental setup**.

In this study, subjects were asked to perform an overt speech production task in which stimuli consisted of well-known political speeches or nursery rhymes ranging between 109 and 411 words in length. The stimulus text was presented visually and scrolled across a computer screen from the right to the left at a constant rate and subjects repeated each word as it appeared on the screen. The rate was set for each subject to be appropriate for the subject's level of attentiveness, cognitive, and comprehension abilities (see Table 1). The computer screen was placed approximately 1 m from the subjects. A single experimental run consisted of reading an entire stimulus passage, and subjects completed between 2–4 runs. All subjects completed the experiment in a single session except for Subject D, who required two sessions. Data collection from the g.USBamp acquisition devices, as well as control of the experimental paradigm were accomplished simultaneously using BCI2000 software (Schalk et al., 2004; Mellinger and Schalk, 2010). A schematic illustrating the experimental setup is shown in (Figure 2).

### 2.3. Signal processing and analysis

The goal of our study was to identify those locations or times in which differential ECoG activity was found between overtly produced speech utterances based on articulatory-acoustic and phonological features (e.g., segmental features) of phonemes^1^. In this work, we used a vowel vs. consonant contrast as the primary phonological discriminatory dimension. In addition, we examined the articulatory-acoustic dimension by testing the manner (e.g., voicing quality: obstruent vs. sonorant) and the place (e.g., location of articulatory closure or constriction) of speech articulation, and voicing (e.g., quasiperiodic oscillations of the vocal folds: voiced vs. voiceless). The place features are primarily used to characterize consonant sounds, while the manner and voicing features can be used in both consonant and vowel descriptions. We conducted an analysis of a feature representing the tongue configurations involved in the production of vowel sounds (e.g., height & frontness within the oral cavity); however, it did not reveal different patterns of spatiotemporal activations and will not be discussed in subsequent sections.

#### 2.3.1. Articulatory-acoustic feature descriptions

The articulatory features used in the present study generally characterize the vocal tract movements and configurations required for speech production. The place of articulation defines a location where speech articulators either close or constrict the vocal tract. In our analysis, high-level descriptions of place of articulation broadly describe the closure of the lips (labial) and the location in the oral cavity where the tongue contacts or approaches the hard and soft palates (coronal and dorsal) (Hall, 2007). The manner of articulation describes the relative closure of the vocal tract and resultant airflow path during phonation; it can be coarsely grouped into obstruents (those articulations that impede airflow in the vocal tract) and sonorants (those which maintain an open vocal tract) (Hall, 2007). The voicing feature indicates whether the vocal folds are active and oscillating during production of speech sounds. Speech sounds are classified as “voiced” if the vocal folds are oscillating and “voiceless” if they are not. All sonorant sounds, including all vowels, in English are considered voiced (with only a few exceptions) while obstruents have voiced and voiceless pairs (e.g., the bilabial pair “b” [voiced] and “p” [voiceless]).

Both the place and manner of articulation can be specified at increasingly refined levels. For place, some examples of the labial feature includes bilabials (“b”) and labiodentals (“v”), an example of a coronal includes alveolars (“d” in “dog”) and palatals, and the dorsal group includes consonants with contact on the velum or soft palate (“g” in “good”). Additionally, the dorsal group can be used to describe the relative movements of all the vowels, though not their specific configurations. These additional place descriptors can further refine the locations of the hard and soft palates contacted by the tongue and vice versa as well (e.g., they describe the portions of the tongue used to contact the palate). The manner of articulation can also be described with finer levels of detail, with examples of the obstruent category including features for stops (“b” in “boy”), fricatives (“v” in “vast”), and affricates (“ch” in “chest”) while the sonorant category contains the features for approximants (“l” in “less”) and nasals (“n” in “nine”). These additional levels of description characterize specific differences in airflow resulting from speech production. To simplify the analysis and provide sufficient data for estimation of our machine learning models, we concentrated on the high-level categorical groupings: obstruent vs. sonorant for manner of articulation, and labial vs. coronal vs. dorsal for place of articulation. A summary of the phonetic feature descriptions used in this study can be found in Table 2.

**Table 2:** **Features and frequencies observed in the speech stimuli**.

**Place of articulation**			**Manner of articulation**		
**Feature**	**Frequency (%)**	**Phonemes**	**Feature**	**Frequency (%)**	**Phonemes**
Labial	22.9	/b p f v m w/	Obstruent	59.1	/b p g k d t f v/
					/tʃ d_Ʒ_ ð θ s z ʃ _Ʒ_/
Coronal	78.0	/t d θ ð s z ʃ _Ʒ_ n/	Sonorant	37.5	/i I ε æ ɑ Ə u ℧/
		/tʃ d_Ʒ_ r l j/			/3^˞^ aI eI a℧ o℧ ᴐI/
					/w j r l m n ŋ/
Dorsal	12.4	/k g w/			
**Phonological**			**Voicing**		
**Feature**	**Frequency (%)**	#	**Feature**	**Frequency (%)**	**Phonemes**
Consonant	60.8	24	Voiced	78.0	/i I ε æ ɑ Ə u ℧/
					/3^˞^ aI eI a℧ o℧ ᴐI/
					/w j r l m n ŋ/
					/b d g v ʃ _Ʒ_ d_Ʒ_/
Vowel	39.2	15	Voiceless	22.0	/p t k f s z tʃ/

#### 2.3.2. Speech segmentation into phonemes

We first segmented the acoustic speech signals into individual phonemes. This segmentation served to (1) separate each individual spoken word and (2) identify and temporally locate phonemes within each word. Our segmentation procedure obtained phonetic transcriptions using a semi-automated algorithm that first isolated the spoken words from silence followed by identification of constituent phonemes. The onset and termination of spoken words were manually located in the audio signal waveforms. Initial manual segmentation of word boundaries was necessary for accurate speech analysis, and was often completed with minimal effort. Following word segmentation, phonemes were automatically labeled and aligned to the audio signal, using a Hidden Markov Model (HMM) classifier with Mel-Frequency Cepstrum Coefficients (MFCC) and their first and second derivatives as features (Rabiner and Juang, 1993). The phonetic transcription and alignment was performed using the HMM ToolKit (HTK) (Young et al., 2006). Our rationale for automated phonetic transcription was to minimize human errors and provide an objective solution for a fair comparison between participants. Each phoneme was then classified as (1) a consonant or a vowel [phonological], (2) an obstruent or sonorant [manner], (3) according to vocal tract contacts or constrictions [place] and (4) voicing status [voicing].

**Figure 3:**
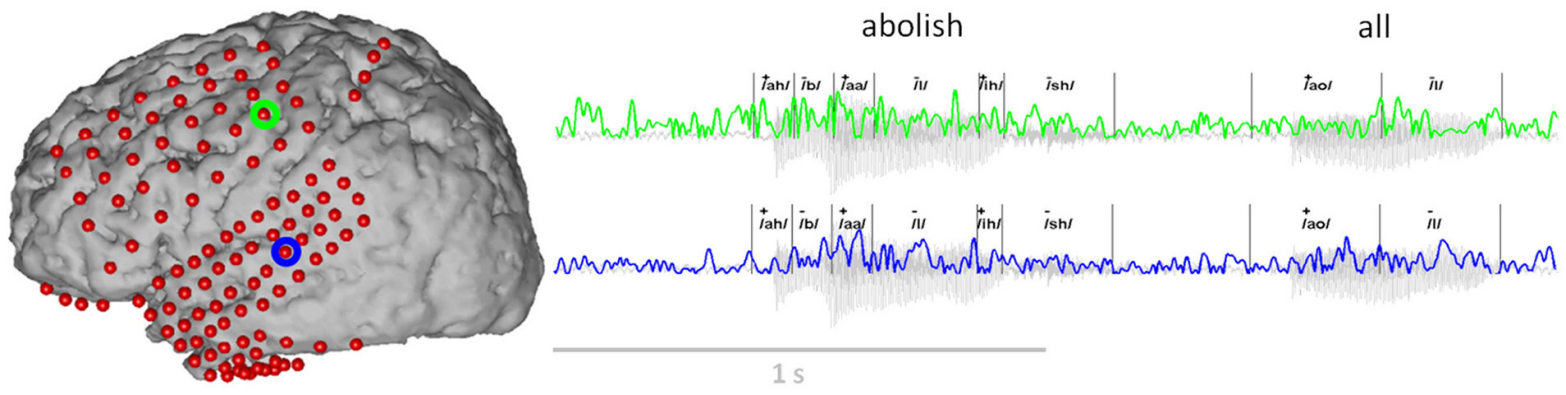
**Example of the ECoG gamma envelope from the two electrodes circled in green and blue, for the production and perception of the words “abolish all.”** The transcription of these two words into phonemes is all also provided, together with the corresponding class label for the articulatory feature “vowel” (“+”: the phoneme is a vowel, “−”: the phoneme is a consonant).

A summary of all phoneme transcriptions and data features used in this study is provided in Table 2. Each speech feature was assigned in a binary fashion in which “+” indicated the presence of a feature, and “−” the absence. Importantly, while the features were coded as binary, any one phoneme may code for multiple combinations of features (e.g., consonant+, obstruent+, labial+ and voicing+ for the “b” sound). In other words, a particular phonemic feature was assigned a value (“+” or “−”) for each phoneme. Overall, we identified 33 different phonemes with 1226–4872 combined occurrences per subject. Each phoneme was defined by a particular onset and offset time that was used for subsequent neurophysiological analyses. An example of audio signal transcription and feature labeling (for the feature: vowel) is given in Figure 3 along with synchronized ECoG recordings (gamma band power) at two electrode sites.

The automatic speech recognition system described above was adapted from the original implementation to achieve robust and accurate speaker-dependent classification for use with all of our study participants. The classifier was first trained on an “ideal” source based on a triphone acoustic model to establish a baseline. Then, the classifier was adapted to account for each participant's individual speech acoustic characteristics using the speech recorded from each subject, creating a speaker-dependent recognition and phonetic transcription system. The speaker-dependent model outperformed the speaker-independent model in terms of producing more accurate phoneme boundaries. All automatic phoneme alignments were visually checked by a speech recognition expert who confirmed their quality.

#### 2.3.3. ECoG segments extraction and labeling

We analyzed event-related changes in 700 ms ECoG epochs aligned to phoneme acoustic onset. To do this, we first high-pass filtered the continuous ECoG recordings using a cutoff frequency of 0.5 Hz and a forward-backward Butterworth filter of order 4 to remove DC signal components (Matlab functions filtfilt and butter). The data were then notch-filtered at 120 Hz using a forward-backward infinite impulse response (IIR) notch filter with a Q-factor of 35 (*q* = ω_0_/bw, where ω_0_ = 120 and *q* = 35) (Antoniou, 1993) to remove the power line harmonics (first harmonic) interference. Note that we did not filter the signals at the fundamental frequency of the power line (60 Hz) nor its other harmonics (180, 240 Hz, etc.) since our analysis only involved the gamma band (70–170 Hz) of the ECoG signals. Following filtering, the ECoG signals were re-referenced to the common average reference (CAR), separately for each grid of implanted electrodes^2^. Finally, the ECoG gamma band power was obtained by applying a bandpass filter in the range of 70–170 Hz using a fourth order forward-backward Butterworth filter, squaring the result and log-transforming the signal.

After preprocessing the recorded ECoG signals, we extracted a 700 ms window of data from the continuous recording. This window was aligned to the onset of each phoneme identified by the semi-automated phoneme transcription procedure described above. Each window was centered on the phoneme onset, and thus consisted of a 350 ms pre-phoneme interval and a 350 ms post-phoneme interval, which provides sufficient opportunity to examine the neurological processing per phoneme. Each window was tagged with the phoneme's feature vector (i.e., “+” or “−” definition for each phonemic feature) for subsequent classification / discrimination analysis.

#### 2.3.4. Classification analysis technique

In the following sections, we describe the method used to evaluate the spatial and temporal patterns of neurological activity involved in speech production. Specifically, we employed a classification analysis to determine which brain regions differ in their patterns of activity during the production of speech that varies by place of articulation, manner of articulation, voicing and phonological category of consonant or vowel (Section 2.3.6). We include also a classification analysis of brain activity during active speaking vs. silence (Section 2.3.5). The same procedure was used for all classification analyses, and is summarized as follows:

1. Process and segment speech signal for features of interest (e.g., speech vs. silence, place, manner and voicing features, phonological features).
2. Preprocess ECoG gamma band power (as in Section 2.3.3).
3. Choose analysis features based on the number of ECoG electrodes, and reduce feature dimensionality according to the minimal Redundancy Maximal Relevance (mRMR) feature selection procedure (Peng et al., 2005).
4. Train and apply a regularized linear discriminant analysis (LDA) classifier (Lotte et al., 2007) for distinguishing selected features using 5 fold cross-validation for each subject and run. Note that feature selection was performed, for each fold of the cross-validation, on the training data only.
5. Evaluate classifier using receiver operating characteristics (ROC) curves, and obtain the area under the curve (AUC) as the primary performance measure.

LDA regularization was achieved using covariance matrix shrinkage according to the Ledoit and Wolf method for automatically estimating large dimensional covariance matrices from small data observations (Ledoit and Wolf, 2004). Regularized LDA using this technique has been previously used in brain-machine interfacing experiments where data and feature dimensionality are consistently problematic (Lotte and Guan, 2010; Blankertz et al., 2011). According to our cross-validation procedure, the data were split into five non-overlapping subsets, four of which were used for LDA training and feature selection and the remaining, mutually-exclusive data set, used for testing. The training and testing procedures were repeated five times, once for each mutually exclusive validation set, and the performance was averaged over all test-set results. Note, classifier training and feature selection were performed only on the training part of each cross-validation fold.

Additionally, we chose area under the ROC curve as the measure of performance since it is specifically designed for unbalanced binary classification problems (Fawcett, 2006). In our study, the number of phonemes labeled “+” for a speech feature was not necessarily the same as the number of phonemes labeled “−,” therefore the classification problem was unbalanced. The “+” class was used as the positive class for ROC curves computation. Statistical significance of the obtained AUC values was determined using the Hanley and McNeil formula for estimating standard error (Hanley and McNeil, 1983). The resulting *p*-value was then corrected for multiple comparisons (number of subjects × number of ECoG electrodes per subject) using the false discovery rate (FDR) approach (Noble, 2009).

#### 2.3.5. Subject screening and inclusion

As a screening measure, we first determined which of the subjects produced ECoG signals that were different between spoken words and silence. Subjects whose classification results exceeded our threshold (see below for details) were analyzed further for the speech feature analysis. According to the classification procedure described in Section 2.3.4, we first manually obtained the boundaries of all words from the acoustic signal and extracted ECoG gamma band power from a 700 ms window centered on each word. We then obtained an equal number of ECoG segments taken from 700 ms windows of silence and labeled the segments as “speech” or “silence.” For each electrode, the pre-processed 700 ms ECoG signal was segmented in time using 50 ms long windows with 25 ms overlap based on the parameters from prior studies (Pei et al., 2011a,b). This procedure resulted in an initial set of 27 gamma-band features per electrode (between 83 and 112 electrodes per subject), which were taken from cortical areas covering the perisylvian and Rolandic cortices (e.g., primary motor, premotor, auditory and somatosensory cortices; Broca's and Wernicke's areas). We then used the mRMR procedure to reduce the feature dimension by selecting 50 features from the larger data set. Last, we obtained the ROC curve and set a threshold of *AUC* > 0.8 for inclusion in the remainder of the speech feature analysis. An *AUC* of 0.5 represents chance performance, we therefore utilized a higher threshold for use as a screening criterion.

#### 2.3.6. Classification of articulatory features

Determination of the differential neurological activity used in the production of each articulatory-acoustic and phonological features (described in Section 2.3.1) was split into separate analyses of spatial topography and temporal dynamics. In the spatial topography analysis, we projected the results onto the cortical surface and plotted the results over time for the temporal dynamics analysis. In these two procedures, the spatial analysis considered ECoG activity at each location throughout each windowed epoch; the temporal analysis considered ECoG activity at a particular time but across all locations.

***2.3.6.1. Spatial topography analysis***. Using the classification procedure described in Section 2.3.4 as a guide, we first obtained the boundaries of all phonemes in the acoustic signal (see Section 2.3.2), extracted the ECoG gamma band power from a 700 ms window centered on the onset of each phoneme, and segmented it in time using 50 ms long windows (25 ms overlap). We then used the mRMR procedure to select 10 time segments per phoneme and electrode to minimize the effects of overfitting while training the regularized LDA classifier. A new classifier was trained on each of the speech features to discriminate between the “+” and “−” category members. To analyze the three levels place of articulation features, we computed three binary comparisons: labial+ vs. labial–, coronal+ vs. coronal− and dorsal+ vs. dorsal−. All other features contained only two levels, therefore, only a single binary comparison is needed for each. We then computed an “activation index” that was proportional to the AUC *p*-value for each tested feature. The activation index (AI) was defined as:

(1) $$\psi (p)=\left\{ \begin{array}{l} -log(p)\;p<0.01\\ 0\;otherwise \end{array}\right.$$

where *log* denotes the natural logarithm. These activation indices for each electrode channel were accumulated across subjects and mapped onto a template brain (Montreal Neurological Institute [MNI]; http://www.bic.mni.mcgill.ca) using in-house Matlab routines (Kubanek and Schalk, 2014).

***2.3.6.2. Temporal dynamics analysis***. The temporal analysis of speech features over the duration of each data segment involved similar processing steps used in the spatial analysis. For each subject, we first limited the temporal analysis to ECoG electrode channels with statistically significant activation indices found in the spatial topography analysis. For this analysis, we first re-estimated the ECoG gamma band power using 50 ms time bins, but with 40 ms overlaps (10 ms steps) for use in the LDA procedure. The change in overlap was used to visualize and analyze the activation index time course with a higher resolution, such resolution is neither needed nor desired for the spatial topography analysis. The same speech features and phonetic boundaries used in the spatial analysis were used here as well. Also in this analysis, dimension reduction and regularization were not required since there was only one data feature (time-binned ECoG band power) per classification attempt. The average AUC was then used to compute a significance *p*-value, corrected for multiple comparisons (subjects, time bins and electrodes with statistically significant activation indices in the spatial topography analysis) using the FDR, and transformed into an activation index. The temporal profiles of the activation indices were averaged across subjects and over all electrodes per speech feature to represent the gross cortical processes involved in the discrimination of speech articulation features.

## 3. Results

### 3.1. Speech vs. silence

We employed a functional screening criteria based on classification results for a speech vs. silence discrimination analysis. These results are summarized in Table 3. Those subjects that did not have neural responses that consistently responded to the task, and thus had signals that could differentiate between speech and silence, were excluded from the remainder of the speech feature analysis. Recall, an AUC value of 0.5 represents chance discrimination, while a value of 1.0 indicates perfect discrimination. None of our analyses resulted in AUC values less than 0.5, indicating that all classifications were above chance levels. However, as illustrated in Table 3, our analysis was not able to well-differentiate the neural activation patterns for the speech vs. silence contrast for subjects A, C, and D using our higher screening threshold (*AUC* < 0.8), which would lead to similarly poor results in any subsequent analyses of articulatory and phonological features. In contrast, the analysis for subjects B, E, F, and G resulted in relatively good differentiation between speech and silence (*AUC* > 0.8). Thus, we included only data from these subjects in the remainder of our study. The resulting combined electrode locations for these four subjects can be found in the bottom right of Figure 1.

**Table 3:** **AUC cross-validation performances obtained for each subject to classify “spoken word” vs. “silence” ECoG segments**.

**Subject**	**A**	**B**	**C**	**D**	**E**	**F**	**G**
AUC	0.57	0.81	0.51	0.68	0.91	0.87	0.91

### 3.2. Cortical mappings and temporal profiles

Topographical cortical mappings and temporal profiles reported here reflect electrodes, grouped over all four subjects, with statistically significant differences in ECoG recordings between our speech features of interest. In our method, each discrimination is along a binary feature dimension and represents a comparison of neural patterns of activation between pairs of speech features.

#### 3.2.1. Place of articulation

**Figure 4:**
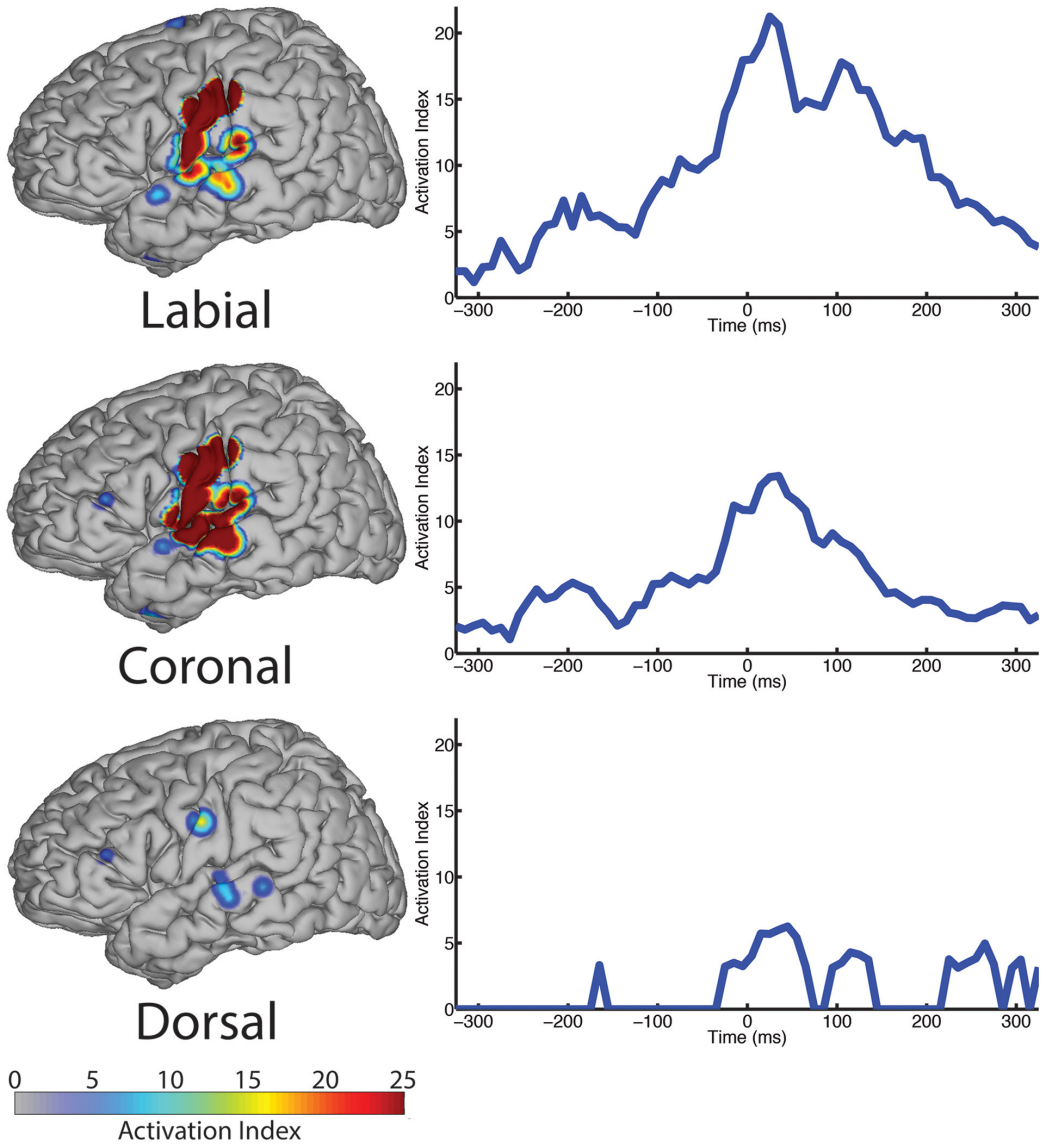
**The spatial topography and temporal dynamics are shown in the left and right columns, respectively, for electrode locations with significant machine learning classification for the “place” category levels: labial, coronal, and dorsal**.

We analyzed ECoG recordings to identify differential neural activity for three place of articulatory features: labial, coronal, and dorsal representing vocal-tract closures at the lips (labial), tongue tip and blade (coronal), and tongue dorsum (dorsal). We then used statistically significant, above-chance LDA classifications (*AUC* > 0.5) as a measure of differential neurological representations of each speech feature. We generally found statistically significant responses across the sensorimotor speech production network and auditory feedback processing regions (see left column Figure 4). The responses superior to the Sylvian fissure are distributed over the primary motor and somatosensory cortices (sensorimotor cortex for speech), while the responses in the temporal lobe are found in perisylvian auditory cortex, particularly in the posterior aspects of the superior temporal gyrus (e.g., Wernicke's area). The coronal feature resulted in the largest topographical montage of statistically significant ECoG electrodes contributing to differentiation of place of articulation (*N* = 19 of 401 electrodes), followed by the labial (*N* = 9) and dorsal (*N* = 3) features. A summary of these results is found in Table 4.

**Table 4:** **Summary of results for place of articulation over all sampled electrodes**.

**Place**	**# Electrodes**	**Peak AI**	**Peak latency**	**Local maxima**
Labial	9	21.24	25 ms	−185, −75, +105 ms
Coronal	19	13.42	35 ms	−195, −85, +95 ms
Dorsal	3	6.25	45 ms	−165, +115 ms

In the temporal dimension group analysis, we found the latency of peak AI for all three place conditions near the onset of phoneme alignment at 0 ms (see Figure 4, right column and summarized in Table 4). Specifically, the labial condition is characterized by an overall difference from all other features that rose markedly to a peak response at +25 ms (with 21.24 peak activation index) and persisted well afterward. The peak activation index for the coronal condition was 13.42 at +35 ms latency and the dorsal condition was 6.25 at +45 ms latency. In general, both the labial and coronal temporal profiles indicated prolonged duration of statistically significant activation indices preceding and following peak response near 25–35 ms while the dorsal condition was much more narrow in its response. We should note that this may be due to the relatively few sounds with constriction or closure of the tongue along soft palate compared to those in the anterior portions of the oral cavity. Furthermore, each of the three place conditions had multiple local maxima throughout the analysis window. Specifically, local maxima were found for the labial condition at −185, −75, and +100 ms, the coronal condition at −195, −85, and +100 ms, and the dorsal condition at −165 and +115 ms.

#### 3.2.2. Manner of articulation

The analysis of place of articulation is oriented toward the articulations and points-of-contact in the oral portion of the upper vocal tract. In contrast, manner of articulation, which describes airflow resulting from constriction or closure (release) is oriented generally as the muscular activation of the entire upper vocal tract (larynx, velum and oral structures). In typical definitions of manner of articulation, categorical features are used to describe the overall airflow. In the present analysis, we follow this convention and examined two main classes of manner: obstruents and sonorants.

**Figure 5:**
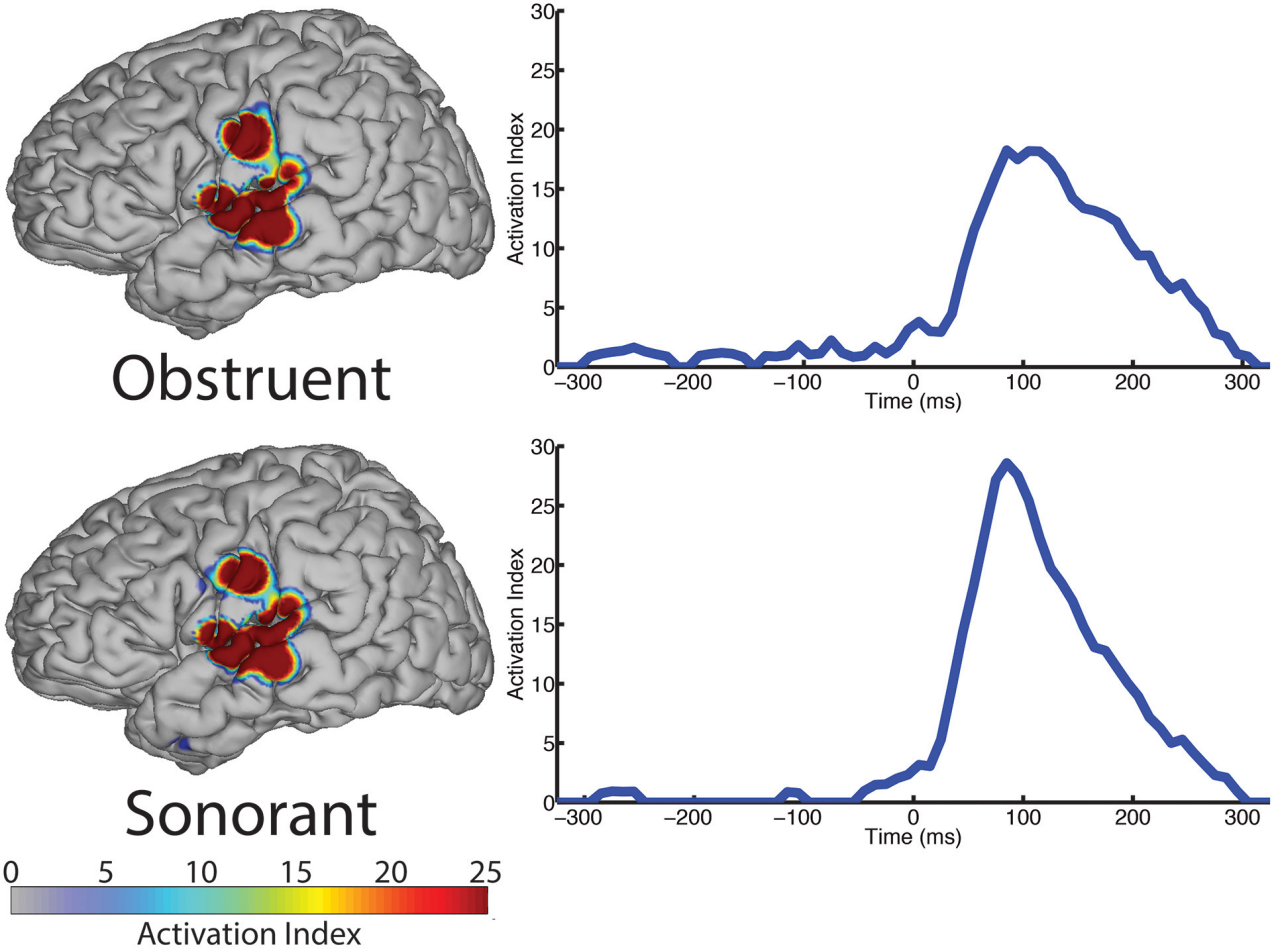
**The spatial topography and temporal dynamics are shown in the left and right columns, respectively, for electrode locations with significant machine learning classification for the “manner” category levels: obstruent and sonorant**.

The spatial topography of electrodes with differential activity patterns between the two manner categories are shown in Figure 5, left column. This analysis revealed statistically significant perisylvian auditory cortex and sensorimotor cortex response contributing to differentiation of the obstruent (*N* = 10 electrodes) and sonorant (*N* = 11 electrodes) features.

The temporal profile results (right column, Figure 5) indicate very limited differences between manner categories prior to phoneme onset (speech-leading latencies with negative intervals) and greater differences at speech-following latencies (positive intervals). Specifically, the peak statistical significance for differentiating manner features from each other at +85 ms for both obstruents and sonorants. These differences are largely present during the entire post-onset speech period. These results are summarized in Table 5.

**Table 5:** **Summary of results for manner of articulation over all sampled electrodes**.

**Manner**	**# Electrodes**	**Peak AI**	**Peak latency**
Obstruent	10	18.26	85 ms
Sonorant	11	28.58	85 ms

#### 3.2.3. Voicing

**Figure 6:**
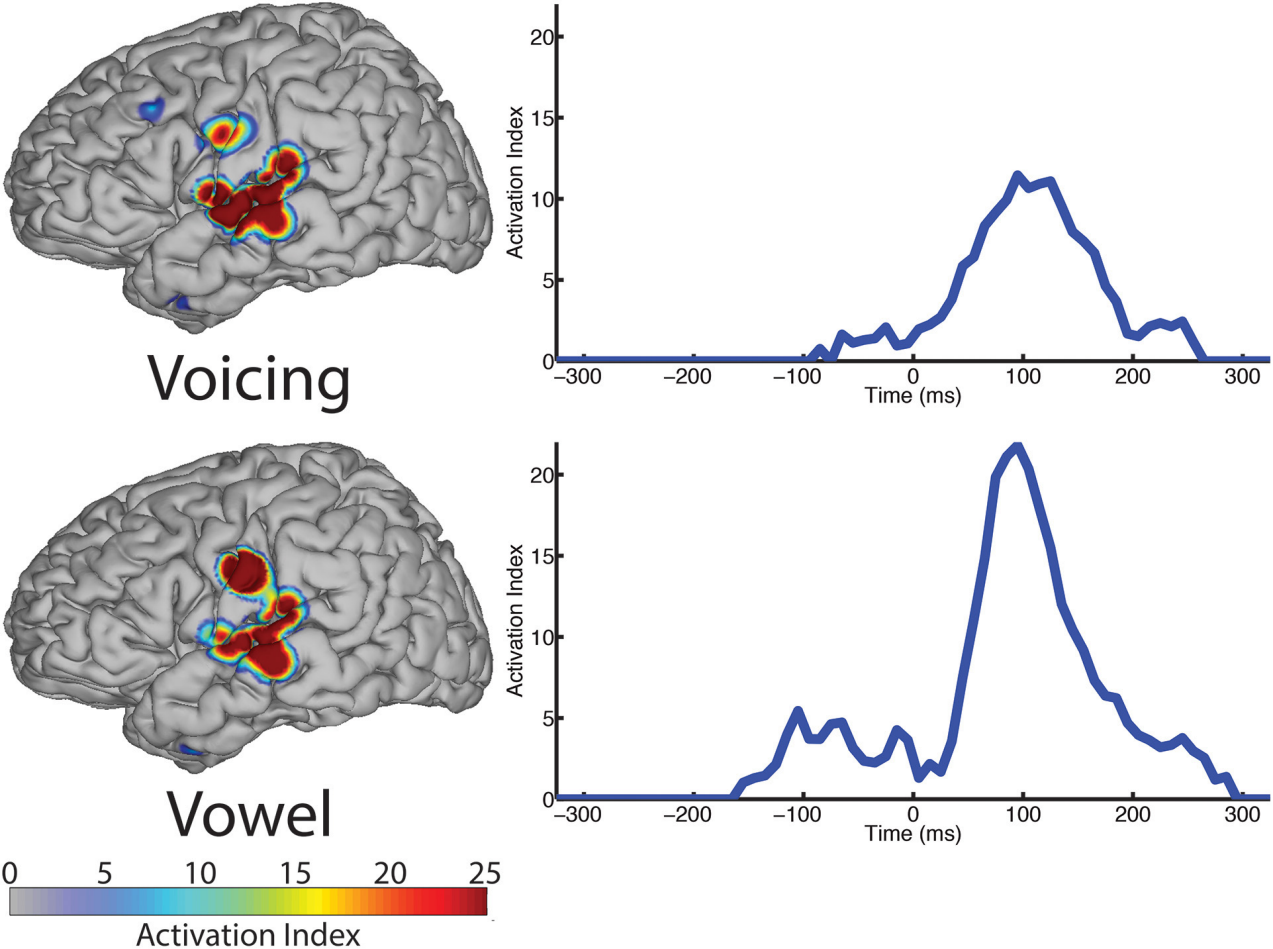
**The spatial topography and temporal dynamics are shown in the left and right columns, respectively, for electrode locations with significant machine learning classification for the “voicing” (i.e., voiced vs. voiceless) and “phonological” (i.e., consonant vs. vowel) categories**.

In contrast to both the manner and place features, voicing refers to only one articulatory structure, the larynx, or more specifically, the vocal folds. The spatial topography of electrodes (left column, Figure 6) with differential patterns of activity between the voiced and voiceless classes of phonemes is concentrated in the perisylvian auditory and motor cortex, with additional activation of the ventral motor cortex. In our analysis, 12 electrodes contributed to differentiation of phonemes along the voicing dimension. The temporal profile of these activations (right column, Figure 6) indicate a peak statistical difference at +95 ms with an activation index of 11.45. There was a smaller local peak just prior to vocalization onset at −25 ms. These results are summarized in Table 6.

**Table 6:** **Summary of results for voicing and phonological category (vowels only) over all sampled electrodes**.

**Manner**	**# Electrodes**	**Peak AI**	**Peak latency**	**Local maxima**
Voicing	12	11.45	95 ms	–25 ms
Vowels	8	21.80	95 ms	–105 ms

#### 3.2.4. Vowel vs. consonant

We examined the vowel vs. consonant contrast to determine whether differences existed in neural activation patterns between production of sounds varying in phonological class. The spatial topography and temporal dynamics representing differences between these two classes were represented by a large region of auditory cortex and a more focal region of sensorimotor cortex. The temporal patterns of neural activation had peak statistical difference +95 ms, but appear to also show moderate differentiation at speech-leading intervals, with a local maxima at −105 ms as shown in Figure 6 (right column). A summary of these results can be found in Table 6.

## 4. Discussion

### 4.1. General comments

In this paper, we identified patterns of cortical topographies and temporal dynamics involved in speech production based on segmental articulatory-acoustic and phonological characteristics. To do this, we used a classification analysis to identify spatial or temporal neurological activity that best discriminated between common sets of articulatory and phonological features of continuous speech production. Some recent studies of speech production using ECoG and intracortical microelectrode recordings have also examined phonetic content (Blakely et al., 2008), and articulatory-acoustic features (Brumberg et al., 2011; Bouchard et al., 2013). Importantly, our task and analyses differ from these earlier attempts by first considering fluent, continuous speech production of whole sentences and paragraphs, which is more natural than isolated utterances and may account for effects of coarticulation. Second, our signal recordings come from a much larger area of the cortical surface, which enabled us to investigate all of the lateral (perisylvian) regions involved in the motor, perceptual and planning neurological processing components of speech production. Last, our analysis focuses on the determination of the neurological activity that differentiates speech segments (e.g., phonemes) from one another based on their phonological and articulatory features.

The continuous speaking task is doubly advantageous as it allows for acquisition of a large amount of phoneme data in a short amount of time, which is imperative when interacting with patients with an ECoG implant. We are also able to analyze simultaneously overlapping processes of phonological processing, execution of articulatory plans and monitoring of acoustic feedback in a manner. Our technique of machine learning classification for discrimination of speech features via ECoG recordings enable direct inference of the neurological structures and dynamics that dissociate production of phonemes with varying phonological and articulatory characteristics. We discuss the major implications of our results along these themes in the following sections. In general, the neurological structures and dynamics revealed in our study overlapped with many of our expectations (Penfield and Roberts, 1959; Bouchard et al., 2013), but our specific analyses identified some striking differences from prior work.

### 4.2. Motor and sensory processing

Speech articulation is composed of at least two “first-order" processes: motor control and sensory (i.e., acoustic) feedback, whose functionality is typically reflected by neural activation of the precentral gyrus and superior temporal gyrus, respectively. Though both types of processes are certainly involved in speech production, the relative timing of neural activations, before or following speech, can help to determine whether processing is related to planning and execution of speech sounds (speech-leading) or feedback maintenance (speech-lagging).

The design of our analysis procedures allowed us to simultaneously analyze neural recordings of continuous speech production from two separate perspectives. In the *place* and *voicing* analyses, we examine the contribution of neural signals to specific articulatory gestures (just the larynx in the case of voicing), while in the *manner* analysis, the motor response is not differentiated. Without examining both, we would have limited the explanatory potential of the recorded data and miss the observation of a dual-role played by sensory cortex (receptive cortex) in speech production. These results are described in more detail in the following sections.

#### 4.2.1. Place of articulation

Place of articulation is easily interpreted along motor and somatosensory dimensions. The placement of a vocal tract closure or constriction necessarily involves movement of the speech articulators as well as tactile (for closure) and proprioceptive (for constriction) somatosensation. In our analysis, we used the place features labial, coronal and dorsal for discriminating ECoG responses as a result of speech articulation. The sensorimotor interpretation for the labial feature refers to closure of the lips, either against each other (bilabial) or of the lower lip against the maxillary teeth (labiodental), both result from the movement of the lip(s) and/or jaw. Similarly, the sensory interpretation for the feature coronal refers to closures occurring between the tongue, maxilla and hard palate, while the motor interpretation refers to muscular involvement of the tongue tip, tongue body and anterior portions of the tongue body as they contact the teeth (dentals), alveolar ridge (alveolars) and hard palate (palatals). Finally, the sensorimotor interpretation of the dorsal feature refers to a vertical and posterior movement of the tongue dorsum for closure against the soft palate, or velum, resulting in the class of velar sounds. Additionally, vowel sounds can be included in the dorsal feature owing to the motor execution requirements of the tongue, but they are not included in any other place category (Hall, 2007), and we do not include them here.

Our analysis revealed a network of neurological structures typically involved in speech motor control with auditory feedback exhibiting patterns of ECoG recordings between three top-level place of articulation categories (labial, coronal, dorsal). These regions included speech sensorimotor cortices, premotor cortex, auditory cortex and Wernicke's area. The combined contributions of all electrodes over the 700 ms time window place category discrimination indicates a primary role in instantaneous motor execution and sensory processing as evidenced by peak statistically significant responses near zero ms latency relative to speech output. These networks are also likely involved in planning and feedback processing as shown by statistically significant responses with local AI maxima at speech leading latencies (−300 to 0 ms) and speech lagging latencies (0–300 ms), respectively. The topography over the primary motor and somatosensory cortices in Figure 4 provide neurophysiological evidence to support this intuitive interpretation. Further, the presence of overlapping sensorimotor locations (defined by electrode placements) suggests the primary motor, premotor and somatosensory cortices are all differentially active across various configurations of the lips and tongue used in speech. The spatial topography also includes perisylvian auditory regions for all feature categories. We interpret these results as representing both prediction of sensory consequences as well as self-perception of vocalized output (e.g., efference motor copy Houde et al., 2002), evidenced by significant contributions preceding and following speech onset, respectively. Like the motor production results, the overlapping auditory cortical responses between conditions indicate that phonemes yield differential ECoG signals during auditory feedback (cf. Pasley et al., 2012).

#### 4.2.2. Manner of articulation

Like place, the manner of articulation also results from muscular contraction of the vocal tract, but is used to describe the quality of vocal airflow during speech production. In the present analysis, we focus on two major feature descriptions of phonemes: obstruent and sonorant. Obstruent sounds are characterized by a blockage of the oral cavity that prohibits sustained voicing, while sonorants facilitate sustained voicing through a relatively open vocal tract. Obstruents include stops (/b/), fricatives (/f/) and affricates (/tʃ/) while sonorants include nasals (/m/), liquids (/l/), glides (/w/) and vowels. It is possible to examine neurological responses to each of the manner subtypes. However, for this analysis we chose to focus on the top-level categories to boost the feature sample size given our phoneme data taken from continuous speaking of paragraph scripts.

The spatial topography and temporal dynamics of statistically significant differences in neural activity between manner features revealed a network involving the premotor cortex, auditory cortex and the posterior superior temporal gyrus (i.e., Wernicke's area) for obstruent and sonorant features. The perisylvian auditory regions were activated to a larger spatial extent compared to the more focal premotor contribution. The temporal dynamics reach peak levels between 65–145 ms following acoustic output of the phoneme and persists throughout the speech production window (up to 300 ms). These observations of spatial and temporal results have three implications: (1) motor and sensory processes are involved in the production of requisite airflow for different classes of phonemes (obstruents and sonorants), (2) that discriminating auditory feedback of manner is represented over a relatively large region of perisylvian auditory cortex, and (3) the differences in motor production of manner is represented by a focal region of motor cortex.

#### 4.2.3. Voicing

Voicing reflects both the laryngeal muscular contractions needed to configure the larynx for phonation as well as the acoustic perception of phonated speech (i.e., contains vocal fold oscillation). The voicing feature is separated into just two classes, voiced and voiceless, and therefore can be represented in our analysis by a single voicing feature. All of the sonorant sounds used in this analysis are included in the [voiced] feature, as are those obstruents that are produced with vocal fold oscillation (e.g., /b/ and /v/). The remaining obstruents are included in the [voiceless] feature.

The spatial topography analysis revealed a network of perisylvian regions extending into both the motor and auditory cortices, and was similar to the patterns found in the manner condition analysis. The peak response occurred at 95 ms post-vocalization, which suggests that this network is primarily involved in the acoustic perception of voicing in self-produced speech. There is, however, a small pre-vocalization response at −25 ms that may be interpreted as involved in the preparation or execution of laryngeal commands for initiating (voiced), or preventing (voiceless) vocal fold oscillation.

#### 4.2.4. Summary of acoustic-articulatory features

The sensorimotor contribution for discriminating manner of articulation and voicing is subdued and focal compared to responses in the place of articulation analysis. According to the analysis of place, widespread activity over the precentral gyrus was likely related to discriminating the three classes of articulation according to different lip, jaw and tongue configurations. In contrast, the focal sensorimotor response observed in the manner and voicing analyses indicates that there is less overall differential sensorimotor activation between the production of obstruent and sonorant phonemes and those with and without voicing. Interestingly, the location of the *manner* and *voicing* sensorimotor response is similar to a region recently proposed to represent laryngeal muscular activation during phonation (Brown et al., 2008; Simonyan and Horwitz, 2011). The larynx, with the respiratory system, is critical for phonation and generation of acoustic signals in the vocal tract. Our result supports the hypothesis that this region is involved in the planning and execution of laryngeal movements used to separately produce voiced and voiceless speech. That a putative neural correlate of laryngeal excitation may be useful for discrimination of obstruents from sonorants potentially implicates a fundamental role of the larynx for planning and executing different manners of articulation as well. Last, recent evidence has also shown this region responds to auditory processing during perception of music (Potes et al., 2012). These combined observations suggest that portions of the motor cortex may be involved in both motor and auditory processing. With the limited number of subjects meeting our screening criteria, we were unfortunately unable to complete a combined spatio-temporal analysis with the statistical power necessary to precisely determine the role of the sensorimotor activity. Future work with an increased sample size will be required to fully investigate these effects.

### 4.3. Examining phonological discrimination

We last examined differences in ECoG recordings between production of consonants and vowels. The category of vowel vs. consonant is mutually exclusive and binary. As seen in Figure 6 (bottom), portions of the speech production and auditory feedback processing networks are differentially active for production of consonants vs. production of vowels, with similar spatial topography as observed in the analysis of manner and voicing. The similarity between these and our previous manner and voicing results is not surprising, as the consonant-vowel, obstruent-sonorant and voiced-voiceless classes encompass nearly the same distribution of phonemes. The main difference between the two features is that certain sonorants are included as consonants, but not obstruents (e.g., nasals, liquids and glides); similarly, some consonants are included in the voiced category largely consisting of vowels. The consonant-vowel contrast is represented by a primary peak in statistically significant differences in activation indices at +95 ms, with a secondary increase in the range −110 to 0 ms relative to onset of speech output. This bimodal response is different than the observed response for manner and place, and likely reflects the complex motor-sensory dynamics involved in the production of all speech sounds, which are particularly intertwined when considering a higher level, phonological concept. In contrast, the manner feature appears to be solely determined by neural analysis of resulting auditory streams.

### 4.4. More features

In the present study, we examined differential neural representations of high-level articulatory-acoustic (place and manner of articulation) and phonological characteristics during speech production. In particular, we focused on the *places:* labial, coronal and dorsal, the *manners:* obstruent and sonorant and *voicing:* voiced and voiceless. As noted previously, the place and manner factors have additional sublevels of increasing refinement (e.g., bilabial and labiodental for place; nasal and fricative for manner). With the present sample size, and the limited amount of time available with each patient, we were not able to examine these additional features. For those factors that we did test, but did not report (e.g., vowel tongue position), we believe that movements of the tongue for vowels are so common to all production attempts that there were no differentially distinguishing features in the ECoG recordings. For results such as these, prior investigations of the overall neural activations found during speech production adequately describe these processes. Future studies with additional subjects and stimuli may help to pick up where this study leaves off. In particular, new studies may optimize speech stimuli selected for representation of as many phonemes and articulations as possible, while maintaining low user effort requirements. In addition, it is possible that the electrode size and spacing in this study was too coarse to disambiguate the fine distinctions between all possible features of speech articulation (Blakely et al., 2008; Bouchard et al., 2013). Advances in micro-ECoG (Blakely et al., 2008; Kellis et al., 2010) and additional studies employing such preparations should be able to more comprehensively investigate additional features.

### 4.5. Potential applications for brain-computer interfacing

Another guiding principle of this work concerned potential application to a neural speech prosthesis, which can interpret brain activity for generating speech output, or a “brain-to-text” device. Our techniques are directly applicable to a motor-speech brain-computer interface (BCI) as the major observations were all based on machine learning classification of speech sounds, which is alternately known as prediction or decoding. Martin and colleagues (Martin et al., 2014) have recently developed a similar method that attempts to predict actual speech acoustic output from recorded ECoG signals. Our work is distinguished from the Martin et al. technique by the adoption of articulatory gestures as the classification basis as opposed to direct acoustic prediction. However, both methods are advantageous as they limit the required classification dictionary (cf. thousands of words needed for word prediction vs. a dozen of articulatory features or acoustic bases) and offer a generative means for word and sentence prediction. In other words, by classifying or predicting a small set of place, manner, voicing and phonological features, it is possible to represent any phoneme, combinations of phonemes (i.e., syllables, words), or even sentences. By considering continuous speech, our methods are also capable of keeping pace with speaking rates observed during natural communication, which would be a marked advancement in the field of augmentative and alternative communication as well as brain-computer interfacing. In contrast, classifying individual discrete words from brain signals would require a prohibitively large data set to select the correct word from the thousands of words used in language.

## 5. Conclusions

In the present study, we examined speech production in the human brain as a sequence of articulatory movements. These sequences have been alternately proposed in the literature to arise from phonetic transcriptions from phonological representations (e.g., phonemes and syllables) (Levelt et al., 1999; Indefrey and Levelt, 2004; Guenther et al., 2006), or theorized as the basis for speech planning and production (e.g., gestural scores) (Saltzman and Munhall, 1989). The present study brings us closer to resolving this debate by first determining whether fundamental articulatory features are identifiable from electrocorticographic recordings in human subjects. The shift toward articulation changes the paradigm of functional neural analysis toward understanding invariant motor outputs of language and away from abstract representations of speech motor control (e.g., phonemes, syllables and words). The combined analysis of motor sequences and phonological representations will provide the requisite means for confirming or rejecting these two different theories of speech production.

## Author contributions

GS designed the research. AR, PB and AG conducted the research protocol. FL and CG contributed data analysis. JB and GS contributed to the interpretation of the results. FL, JB and GS wrote the paper.

### Conflict of interest statement

The authors declare that the research was conducted in the absence of any commercial or financial relationships that could be construed as a potential conflict of interest.

## Footnotes

^1^As defined by the International Phonetic Association, a phoneme is “the smallest segmental unit of sound employed to form meaningful contrasts between utterances” (International Phonetic Association, 1999).

^2^Most subjects had more than one ECoG grid implanted; therefore, the electrodes from each grid were re-referenced to the grid average.
